# *tert*-Butyl 2-hy­droxy-3-(4-methyl­benzene­sulfonamido)­butano­ate

**DOI:** 10.1107/S1600536811004934

**Published:** 2011-02-19

**Authors:** Mohamed I. Fadlalla, Holger B. Friedrich, Glenn E. M. Maguire, Bernard Omondi

**Affiliations:** a School of Chemistry, University of KwaZulu–Natal, Westville Campus, Private Bag X54001, Durban 4000, South Africa; b Research Centre for Synthesis and Catalysis, Department of Chemistry, University of Johannesburg, PO Box 524 Auckland Park, Johannesburg 2006, South Africa

## Abstract

In the crystal of the title compound, C_15_H_23_NO_5_S, mol­ecules are linked through N—H⋯O and O—H⋯O hydrogen-bond inter­actions, resulting in centrosymmetric dimers in which the N—H⋯O inter­actions generate *R*_2_^2^(12) rings and the O—H⋯O inter­actions generate *R*_2_^2^(14) rings. Weak inter­molecular C—H⋯O inter­actions are also observed.

## Related literature

For related structures of β-amino alcohols, see: Lohray *et al.* (2002 ▶); Bodkin *et al.* (2008 ▶). For the structures of tosyl­amino compounds, see: Coote *et al.* (2008 ▶); Liu *et al.* (2005 ▶); Fadlalla *et al.* (2010 ▶). For the synthesis of the title compound, see: Naicker *et al.* (2008 ▶); Govender *et al.* (2003 ▶). For hydrogen-bond motifs, see: Bernstein *et al.* (1995 ▶).
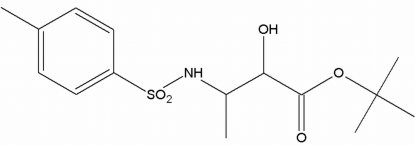


## Experimental

### 

#### Crystal data

C_15_H_23_NO_5_S
*M* *_r_* = 329.4
Triclinic, 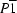

*a* = 9.6038 (8) Å
*b* = 9.9059 (8) Å
*c* = 10.1064 (11) Å
α = 119.342 (2)°
β = 92.307 (2)°
γ = 93.422 (2)°
*V* = 833.95 (13) Å^3^
*Z* = 2
Mo *K*α radiation
μ = 0.22 mm^−1^
*T* = 100 K
0.22 × 0.18 × 0.14 mm

#### Data collection

Bruker X8 APEXII 4K Kappa CCD diffractometer
Absorption correction: multi-scan (*SADABS*; Bruker, 2007 ▶) *T*_min_ = 0.954, *T*_max_ = 0.970
24635 measured reflections
4192 independent reflections
3712 reflections with *I* > 2σ(*I*)
*R*_int_ = 0.031

#### Refinement

*R*[*F* ^2^ > 2σ(*F* ^2^)] = 0.030
*wR*(*F* ^2^) = 0.083
*S* = 1.05
4192 reflections
209 parameters
H atoms treated by a mixture of independent and constrained refinement
Δρ_max_ = 0.45 e Å^−3^
Δρ_min_ = −0.39 e Å^−3^

### 

Data collection: *APEX2* (Bruker, 2007 ▶); cell refinement: *SAINT-Plus* (Bruker, 2007 ▶); data reduction: *SAINT-Plus* and *XPREP* (Bruker, 2007 ▶); program(s) used to solve structure: *SHELXS97* (Sheldrick, 2008 ▶); program(s) used to refine structure: *SHELXL97* (Sheldrick, 2008 ▶); molecular graphics: *DIAMOND* (Brandenburg & Putz, 2005 ▶) and *ORTEP-3* (Farrugia, 1997 ▶); software used to prepare material for publication: *WinGX* (Farrugia, 1999 ▶).

## Supplementary Material

Crystal structure: contains datablocks global, I. DOI: 10.1107/S1600536811004934/hg2795sup1.cif

Structure factors: contains datablocks I. DOI: 10.1107/S1600536811004934/hg2795Isup2.hkl

Additional supplementary materials:  crystallographic information; 3D view; checkCIF report

## Figures and Tables

**Table 1:** Hydrogen-bond geometry (Å, °)

*D*—H⋯*A*	*D*—H	H⋯*A*	*D*⋯*A*	*D*—H⋯*A*
N1—H1*D*⋯O2^i^	0.842 (16)	2.059 (16)	2.8625 (12)	159.5 (14)
O3—H3⋯O4^i^	0.84	2.40	3.2041 (12)	162
C1—H1*C*⋯O4^ii^	0.98	2.54	3.4936 (14)	164

Symmetry codes: (i) 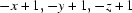; (ii) 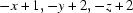.

**Fig. 1.**
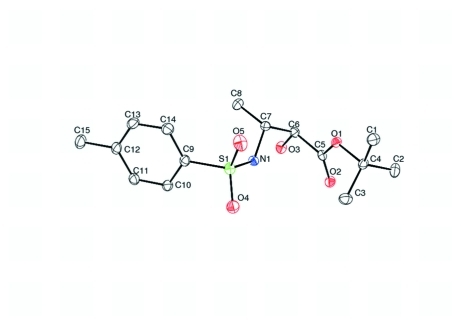
View of (I) (50% probability displacement ellipsoids). H atoms have been omited for clarity.

**Fig. 2.**
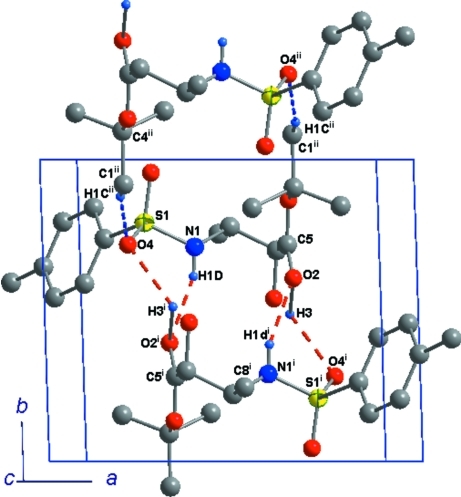
N—H···O and O—H···O hydrogen bond interactions in the crystal structure of (I). [Symmetry operators: (i) = 1 - x, 1 - y, 1 - z; (ii) = 1 - x, 2 - y, 2 - z]

## supplementary crystallographic information

### Comment

The aminohydroxylation reaction of alkenes is the most simple, single step reaction in the production of β-amino alcohols. The product (β-amino alcohol) is present in many natural products and biologically active compounds (such as Acranil which is an antiprotozoal drug) (Bodkin *et al.*, 2008, Lohray *et al.*, 2002). Furthermore, β-amino alcohols are utilized in asymmetric catalysis in the synthesis of chiral ligands. As part of investigating new heterogeneous route to the aminohydroxylation reaction to produce β-amino alcohols, we report the crystal structure of the title compound (I). The molecular structure of (I) is related to that of (2,3)-Methyl 2-hydroxy-3-(4-methylbenzenesulfonamido)-3-phenylpropanoate (Fadlalla *et al.*, (2010). Other related structures have been reported by Coote *et al.* (2008) and Liu *et al.*, (2005).

Fig. I shows the asymetric unit of (I). The compound is chiral and has an S chirality at C6 and an *R* chirality at C7. In the crystal, adjacent molecules are connected by a pair of N—H···O and O—H···O hydrogen bonds (Fig. 2) that result in centrosymmetric dimers that can be described by *R*_2_^2^(12) and *R*_2_^2^(14) graph set notations (Bernstein *et al.* 1995) respectively. In addition, weak C—H···O intermolecular interactions (Table 1) contribute to the stability of the crystal lattice.

### Experimental

The title compound (I) was obtained through a modified literature method (Naicker *et al.*, 2008, Govender *et al.*, 2003).To a nitrogen saturated Schlenk tube 6 ml of a mixture of acetonitrile and water (1:1 *v*/*v*), *tert*-butylcrotonate (76 µ*L*, 0.478 mmol), chloramine-T (0.956 g, 0.956 mmol), hydrotalcite-like catalyst (0.03 g) were added in that order. The catalyst was gravity filtered off after 15 h. The reaction mixture was then washed with sodium sulfite (1 g in 20 ml of de-ionized water) followed by 15 ml of ethyl acetate. The aqueous layer was separated from the organic layer and further washed by 3x 15 ml of ethyl acetate. The solvent of the combined organic mixture was removed *in vacuo*. The resulting crude product was purified by preparative high preasure liquid chromatography to yield the title compound as a white solid. Crystals of I were obtained by slow evaporation of a hexane layered solution of the compound in dichloro methane at room temperature (m.p. 142–145 K).

Spectroscopic data: ^1^H NMR (400 MHz, CDCl_3_, δ. p.p.m.): = 0.9 (d, 3H), 1.5 (s, 9H), 2.4 (s, 3H), 3.2 (d, 1H), 3.8 (m, 2H), 4.7 (d, 1H), 7.3 (d, 2H), 7.7 (d, 2H). ^13^C NMR (100 MHz, CDCl_3_, δ. p.p.m.): = 17.9 (s,1 C), 21.5 (s, 1 C), 27.9 (s, 3 C), 51.5 (s, 1 C), 73.6 (s, 1 C), 84.1 (s, 1 C), 126.9 (s, 2 C), 138.6 (s, 1 C), 143.3 (s, 1 C), 171.6 (s, 1 C). IR (cm^-1^): = 3446 (*m*), (OH), 3260 (*m*), (NH), 2985 (w), 2919 (w), 1598 (w), (ar), 1716 (*m*), (C=O), 1048 (*m*), (S=O). Mass calculated = 329, MS = 351 m/*z* (*M* + Na).

### Refinement

The methyl, methine and aromatic H atoms were placed in geometrically idealized positions and constrained to ride on their parent atoms, with C—H = 0.95 Å and *U*_iso_(H) = 1.2*U*_eq_(C) for aromatic, C—H = 0.98 Å and *U*_iso_(H) = 1.2*U*_eq_(C) for CH_3_, C—H = 1.00 Å and *U*_iso_(H) = 1.2*U*_eq_(C) for CH. N—H = 0.84 Å and *U*_iso_(H) = 1.2*U*_eq_(N) for N—H and O—H = 0.84 Å and *U*_iso_(H) = 1.5*U*_eq_(O).

### Crystal data

**Table:**

C_15_H_23_NO_5_S	*Z* = 2
*M**_r_* = 329.4	*F*(000) = 352
Triclinic, *P*1	*D*_x_ = 1.312 Mg m^−^^3^
Hall symbol: -P 1	Mo *K*α radiation, λ = 0.71073 Å
*a* = 9.6038 (8) Å	Cell parameters from 24635 reflections
*b* = 9.9059 (8) Å	θ = 2.1–28.5°
*c* = 10.1064 (11) Å	µ = 0.22 mm^−^^1^
α = 119.342 (2)°	*T* = 100 K
β = 92.307 (2)°	Block, colourless
γ = 93.422 (2)°	0.22 × 0.18 × 0.14 mm
*V* = 833.95 (13) Å^3^	

### Data collection

**Table:**

Bruker X8 APEXII 4K Kappa CCD diffractometer	3712 reflections with *I* > 2σ(*I*)
graphite	*R*_int_ = 0.031
φ and ω scans	θ_max_ = 28.5°, θ_min_ = 2.1°
Absorption correction: multi-scan (*SADABS*; Bruker, 2007)	*h* = −12→12
*T*_min_ = 0.954, *T*_max_ = 0.970	*k* = −13→13
24635 measured reflections	*l* = −13→13
4192 independent reflections	

### Refinement

**Table:**

Refinement on *F*^2^	Primary atom site location: structure-invariant direct methods
Least-squares matrix: full	Secondary atom site location: difference Fourier map
*R*[*F*^2^ > 2σ(*F*^2^)] = 0.030	Hydrogen site location: inferred from neighbouring sites
*wR*(*F*^2^) = 0.083	H atoms treated by a mixture of independent and constrained refinement
*S* = 1.05	*w* = 1/[σ^2^(*F*_o_^2^) + (0.0395*P*)^2^ + 0.3237*P*] where *P* = (*F*_o_^2^ + 2*F*_c_^2^)/3
4192 reflections	(Δ/σ)_max_ = 0.005
209 parameters	Δρ_max_ = 0.45 e Å^−^^3^
0 restraints	Δρ_min_ = −0.39 e Å^−^^3^

### Special details

**Table:**

Experimental. The intensity data was collected on a Bruker X8 Apex 4 K CCD diffractometer using an exposure time of 15 sec/per frame. A total of 3328 frames were collected with a frame width of 0.5° covering upto θ = 28.45° with 99.8% completeness accomplished.
Geometry. All e.s.d.'s (except the e.s.d. in the dihedral angle between two l.s. planes) are estimated using the full covariance matrix. The cell e.s.d.'s are taken into account individually in the estimation of e.s.d.'s in distances, angles and torsion angles; correlations between e.s.d.'s in cell parameters are only used when they are defined by crystal symmetry. An approximate (isotropic) treatment of cell e.s.d.'s is used for estimating e.s.d.'s involving l.s. planes.
Refinement. Refinement of *F*^2^ against ALL reflections. The weighted *R*-factor *wR* and goodness of fit *S* are based on *F*^2^, conventional *R*-factors *R* are based on *F*, with *F* set to zero for negative *F*^2^. The threshold expression of *F*^2^ > σ(*F*^2^) is used only for calculating *R*-factors(gt) *etc*. and is not relevant to the choice of reflections for refinement. *R*-factors based on *F*^2^ are statistically about twice as large as those based on *F*, and *R*- factors based on ALL data will be even larger.

### Fractional atomic coordinates and isotropic or equivalent isotropic displacement parameters (Å^2^)

**Table:**

	*x*	*y*	*z*	*U*_iso_*/*U*_eq_	
C1	0.74584 (13)	1.08296 (13)	0.93414 (13)	0.0208 (2)	
H1A	0.6562	1.118	0.9193	0.031*	
H1B	0.8195	1.1223	0.8942	0.031*	
H1C	0.7685	1.1225	1.0431	0.031*	
C2	0.87462 (12)	0.84588 (14)	0.86348 (14)	0.0241 (2)	
H2A	0.9444	0.8772	0.8132	0.036*	
H2B	0.8629	0.7322	0.8145	0.036*	
H2C	0.9061	0.8896	0.9712	0.036*	
C3	0.61532 (13)	0.83957 (14)	0.90149 (13)	0.0222 (2)	
H3A	0.6156	0.7262	0.8517	0.033*	
H3B	0.5263	0.8661	0.8736	0.033*	
H3C	0.627	0.8836	1.0122	0.033*	
C4	0.73538 (11)	0.90605 (13)	0.85019 (12)	0.0165 (2)	
C5	0.68030 (10)	0.71782 (12)	0.58068 (12)	0.0145 (2)	
C6	0.63194 (11)	0.69956 (12)	0.42678 (12)	0.0151 (2)	
H6	0.7064	0.7491	0.3939	0.018*	
C7	0.49600 (11)	0.77696 (12)	0.43670 (12)	0.0152 (2)	
H7	0.5149	0.891	0.5094	0.018*	
C8	0.44465 (12)	0.75423 (15)	0.28179 (13)	0.0223 (2)	
H8A	0.3603	0.8085	0.2919	0.033*	
H8B	0.4228	0.643	0.2101	0.033*	
H8C	0.5178	0.7965	0.2438	0.033*	
C9	0.12195 (11)	0.73713 (13)	0.43575 (12)	0.0166 (2)	
C10	0.05152 (11)	0.58888 (13)	0.37062 (13)	0.0177 (2)	
H10	0.0739	0.5198	0.4067	0.021*	
C11	−0.05154 (11)	0.54303 (14)	0.25272 (13)	0.0197 (2)	
H11	−0.0984	0.4414	0.2071	0.024*	
C12	−0.08741 (11)	0.64442 (15)	0.19995 (13)	0.0209 (2)	
C13	−0.01768 (12)	0.79315 (15)	0.26927 (14)	0.0233 (2)	
H13	−0.0425	0.8639	0.2362	0.028*	
C14	0.08753 (12)	0.84021 (14)	0.38584 (14)	0.0215 (2)	
H14	0.1352	0.9413	0.4308	0.026*	
C15	−0.19933 (13)	0.59239 (18)	0.07100 (15)	0.0296 (3)	
H15A	−0.1592	0.5302	−0.0263	0.044*	
H15B	−0.2358	0.6838	0.0733	0.044*	
H15C	−0.2756	0.5295	0.0824	0.044*	
N1	0.39695 (9)	0.71013 (11)	0.50168 (10)	0.01484 (18)	
O1	0.70108 (8)	0.86607 (8)	0.68847 (8)	0.01528 (15)	
O2	0.69788 (8)	0.60683 (9)	0.59739 (9)	0.01913 (17)	
O3	0.60741 (8)	0.53975 (9)	0.31707 (9)	0.01922 (17)	
H3	0.6564	0.4879	0.3431	0.029*	
O4	0.21687 (9)	0.71817 (10)	0.66814 (9)	0.02355 (18)	
O5	0.29082 (9)	0.95624 (10)	0.65376 (10)	0.02641 (19)	
S1	0.25877 (3)	0.79036 (3)	0.58022 (3)	0.01691 (8)	
H1D	0.3859 (16)	0.6124 (18)	0.4581 (17)	0.025 (4)*	

### Atomic displacement parameters (Å^2^)

**Table:**

	*U*^11^	*U*^22^	*U*^33^	*U*^12^	*U*^13^	*U*^23^
C1	0.0283 (6)	0.0164 (5)	0.0150 (5)	−0.0004 (4)	−0.0005 (4)	0.0059 (4)
C2	0.0216 (5)	0.0238 (6)	0.0233 (6)	0.0015 (4)	−0.0067 (4)	0.0095 (5)
C3	0.0261 (6)	0.0222 (5)	0.0196 (5)	0.0003 (4)	0.0039 (4)	0.0114 (5)
C4	0.0196 (5)	0.0169 (5)	0.0124 (5)	0.0001 (4)	−0.0021 (4)	0.0072 (4)
C5	0.0108 (4)	0.0148 (5)	0.0162 (5)	0.0007 (4)	0.0006 (4)	0.0065 (4)
C6	0.0152 (5)	0.0141 (5)	0.0140 (5)	0.0003 (4)	0.0008 (4)	0.0055 (4)
C7	0.0159 (5)	0.0144 (5)	0.0160 (5)	0.0000 (4)	−0.0002 (4)	0.0083 (4)
C8	0.0221 (5)	0.0290 (6)	0.0201 (5)	0.0010 (4)	−0.0021 (4)	0.0159 (5)
C9	0.0138 (5)	0.0186 (5)	0.0178 (5)	0.0042 (4)	0.0029 (4)	0.0088 (4)
C10	0.0153 (5)	0.0201 (5)	0.0206 (5)	0.0044 (4)	0.0031 (4)	0.0119 (4)
C11	0.0152 (5)	0.0230 (5)	0.0211 (5)	0.0016 (4)	0.0018 (4)	0.0112 (5)
C12	0.0135 (5)	0.0337 (6)	0.0211 (5)	0.0060 (4)	0.0051 (4)	0.0171 (5)
C13	0.0196 (5)	0.0310 (6)	0.0306 (6)	0.0089 (5)	0.0064 (5)	0.0229 (5)
C14	0.0192 (5)	0.0197 (5)	0.0287 (6)	0.0046 (4)	0.0042 (4)	0.0140 (5)
C15	0.0204 (6)	0.0507 (8)	0.0263 (6)	0.0044 (5)	0.0003 (5)	0.0256 (6)
N1	0.0145 (4)	0.0121 (4)	0.0174 (4)	0.0014 (3)	0.0016 (3)	0.0068 (4)
O1	0.0187 (4)	0.0130 (3)	0.0129 (3)	0.0001 (3)	−0.0015 (3)	0.0058 (3)
O2	0.0199 (4)	0.0148 (4)	0.0221 (4)	0.0016 (3)	−0.0027 (3)	0.0090 (3)
O3	0.0220 (4)	0.0143 (4)	0.0157 (4)	0.0026 (3)	−0.0009 (3)	0.0031 (3)
O4	0.0227 (4)	0.0316 (5)	0.0163 (4)	0.0011 (3)	0.0028 (3)	0.0118 (4)
O5	0.0257 (4)	0.0159 (4)	0.0268 (4)	0.0034 (3)	0.0006 (3)	0.0022 (3)
S1	0.01644 (13)	0.01627 (13)	0.01483 (13)	0.00257 (9)	0.00163 (9)	0.00506 (10)

### Geometric parameters (Å, °)

**Table:**

C1—C4	1.5218 (15)	C8—H8B	0.98
C1—H1A	0.98	C8—H8C	0.98
C1—H1B	0.98	C9—C14	1.3921 (15)
C1—H1C	0.98	C9—C10	1.3949 (15)
C2—C4	1.5235 (15)	C9—S1	1.7733 (11)
C2—H2A	0.98	C10—C11	1.3881 (15)
C2—H2B	0.98	C10—H10	0.95
C2—H2C	0.98	C11—C12	1.4012 (16)
C3—C4	1.5245 (16)	C11—H11	0.95
C3—H3A	0.98	C12—C13	1.3938 (18)
C3—H3B	0.98	C12—C15	1.5106 (16)
C3—H3C	0.98	C13—C14	1.3914 (17)
C4—O1	1.4978 (12)	C13—H13	0.95
C5—O2	1.2115 (13)	C14—H14	0.95
C5—O1	1.3277 (12)	C15—H15A	0.98
C5—C6	1.5244 (14)	C15—H15B	0.98
C6—O3	1.4161 (12)	C15—H15C	0.98
C6—C7	1.5353 (14)	N1—S1	1.6172 (9)
C6—H6	1	N1—H1D	0.842 (16)
C7—N1	1.4750 (13)	O3—H3	0.84
C7—C8	1.5245 (15)	O4—S1	1.4422 (9)
C7—H7	1	O5—S1	1.4393 (9)
C8—H8A	0.98		
			
C4—C1—H1A	109.5	C7—C8—H8B	109.5
C4—C1—H1B	109.5	H8A—C8—H8B	109.5
H1A—C1—H1B	109.5	C7—C8—H8C	109.5
C4—C1—H1C	109.5	H8A—C8—H8C	109.5
H1A—C1—H1C	109.5	H8B—C8—H8C	109.5
H1B—C1—H1C	109.5	C14—C9—C10	120.57 (10)
C4—C2—H2A	109.5	C14—C9—S1	120.59 (9)
C4—C2—H2B	109.5	C10—C9—S1	118.82 (8)
H2A—C2—H2B	109.5	C11—C10—C9	119.49 (10)
C4—C2—H2C	109.5	C11—C10—H10	120.3
H2A—C2—H2C	109.5	C9—C10—H10	120.3
H2B—C2—H2C	109.5	C10—C11—C12	120.95 (11)
C4—C3—H3A	109.5	C10—C11—H11	119.5
C4—C3—H3B	109.5	C12—C11—H11	119.5
H3A—C3—H3B	109.5	C13—C12—C11	118.43 (10)
C4—C3—H3C	109.5	C13—C12—C15	121.30 (11)
H3A—C3—H3C	109.5	C11—C12—C15	120.27 (11)
H3B—C3—H3C	109.5	C14—C13—C12	121.41 (10)
O1—C4—C1	102.44 (8)	C14—C13—H13	119.3
O1—C4—C2	109.60 (9)	C12—C13—H13	119.3
C1—C4—C2	111.41 (9)	C13—C14—C9	119.13 (11)
O1—C4—C3	108.99 (9)	C13—C14—H14	120.4
C1—C4—C3	111.22 (9)	C9—C14—H14	120.4
C2—C4—C3	112.66 (10)	C12—C15—H15A	109.5
O2—C5—O1	125.85 (10)	C12—C15—H15B	109.5
O2—C5—C6	122.05 (9)	H15A—C15—H15B	109.5
O1—C5—C6	112.09 (9)	C12—C15—H15C	109.5
O3—C6—C5	109.87 (8)	H15A—C15—H15C	109.5
O3—C6—C7	108.58 (8)	H15B—C15—H15C	109.5
C5—C6—C7	110.83 (8)	C7—N1—S1	123.52 (7)
O3—C6—H6	109.2	C7—N1—H1D	116.2 (10)
C5—C6—H6	109.2	S1—N1—H1D	112.5 (10)
C7—C6—H6	109.2	C5—O1—C4	119.50 (8)
N1—C7—C8	114.28 (9)	C6—O3—H3	109.5
N1—C7—C6	105.79 (8)	O5—S1—O4	120.06 (5)
C8—C7—C6	110.98 (9)	O5—S1—N1	107.61 (5)
N1—C7—H7	108.5	O4—S1—N1	105.57 (5)
C8—C7—H7	108.5	O5—S1—C9	108.09 (5)
C6—C7—H7	108.5	O4—S1—C9	106.33 (5)
C7—C8—H8A	109.5	N1—S1—C9	108.80 (5)
			
O2—C5—C6—O3	2.43 (14)	S1—C9—C14—C13	178.17 (9)
O1—C5—C6—O3	−178.31 (8)	C8—C7—N1—S1	−76.81 (11)
O2—C5—C6—C7	122.43 (11)	C6—C7—N1—S1	160.79 (7)
O1—C5—C6—C7	−58.31 (11)	O2—C5—O1—C4	−6.69 (15)
O3—C6—C7—N1	67.16 (10)	C6—C5—O1—C4	174.08 (8)
C5—C6—C7—N1	−53.61 (11)	C1—C4—O1—C5	−178.29 (9)
O3—C6—C7—C8	−57.32 (11)	C2—C4—O1—C5	63.33 (12)
C5—C6—C7—C8	−178.09 (9)	C3—C4—O1—C5	−60.39 (12)
C14—C9—C10—C11	1.46 (16)	C7—N1—S1—O5	−33.34 (10)
S1—C9—C10—C11	−177.12 (8)	C7—N1—S1—O4	−162.69 (8)
C9—C10—C11—C12	−1.07 (17)	C7—N1—S1—C9	83.54 (9)
C10—C11—C12—C13	−0.37 (17)	C14—C9—S1—O5	15.26 (11)
C10—C11—C12—C15	179.70 (10)	C10—C9—S1—O5	−166.16 (9)
C11—C12—C13—C14	1.47 (17)	C14—C9—S1—O4	145.40 (9)
C15—C12—C13—C14	−178.60 (11)	C10—C9—S1—O4	−36.01 (10)
C12—C13—C14—C9	−1.10 (18)	C14—C9—S1—N1	−101.32 (10)
C10—C9—C14—C13	−0.39 (17)	C10—C9—S1—N1	77.26 (9)

### Hydrogen-bond geometry (Å, °)

**Table:**

*D*—H···*A*	*D*—H	H···*A*	*D*···*A*	*D*—H···*A*
N1—H1D···O2^i^	0.842 (16)	2.059 (16)	2.8625 (12)	159.5 (14)
O3—H3···O4^i^	0.84	2.40	3.2041 (12)	162
C1—H1C···O4^ii^	0.98	2.54	3.4936 (14)	164

Symmetry codes: (i) −*x*+1, −*y*+1, −*z*+1; (ii) −*x*+1, −*y*+2, −*z*+2.
